# Anti-Inflammatory Cycloartane-Type Saponins of *Astragalus membranaceus*

**DOI:** 10.3390/molecules18043725

**Published:** 2013-03-25

**Authors:** Dae-Young Lee, Hyung-Jun Noh, Jehun Choi, Kyeong-Hee Lee, Min-Ho Lee, Ji-Hyun Lee, Yoonpyo Hong, Seung-Eun Lee, Seung-Yu Kim, Geum-Soog Kim

**Affiliations:** 1 Department of Herbal Crop Research, National Institute of Horticultural and Herbal Science, RDA, Eumseong 369-873, Korea; 2 Department of Food Technology and Services, Eulji University, Seongnam 461-723, Korea

**Keywords:** *Astragalus membranaceus*, cycloartane-type triterpene, agroastragaloside V, nitric oxide

## Abstract

A new cycloartane-type triterpene glycoside, agroastragaloside V (**1**) was isolated from the roots of *Astragalus membranaceus*. The structure was identified as 3-*O*-*β*-(2'-*O*-acetyl)-d-xylopyranosyl-6-*O*-*β*-d-glucopyranosyl-(24*S*)-3*β*,6*α*,24*α*,25-tetrahydroxy-9,19-cyclolanostane, by means of spectroscopic methods, including HR-FAB/MS, 1D NMR (^1^H, ^13^C, DEPT), 2D NMR (gCOSY, gHSQC, gHMBC, NOESY), and IR spectroscopy. Four known cycloartane glycosides, namely, agroastragaloside I (**2**), agroastragaloside II (**3**), isoastragaloside II (**4**) and astragaloside IV (**5**) were also isolated. All isolated compounds were tested for the ability to inhibit LPS-induced nitric oxide production in RAW264.7 macrophages.

## 1. Introduction

*Astragalus* species are among the most widely distributed in northern temperate regions and tropical African mountains [1]. Five species have been identified in Korea and the primary parts used for medicinal purposes, are cylindrical, but not usually branched, measure around 30–90 cm in length, and are covered with a tough, yellowish-brown skin with a sweet white inner pulp [2]. Radix astragali, the dried root of *Astragalus membranaceus* (FISCH.) BGE., known as Huangqi in China and Korea, is one of the most widely used medicinal herbs prescribed in many Chinese formulas to reinforce “Qi” (the vital energy) [3]. Studies of its pharmacological and clinical uses have demonstrated that Astragali Radix has many biological functions, including hepatoprotection [4], neuroprotection [5], cardiotonic [6], anti-aging activity [7], anti-cancer effects [8], and anti-inflammatory effects [9]. *Astragalus* species are rich in cycloartane-type triterpene glycosides that possess diverse biological activities. Some cycloartane triterpene glycosides have been shown to have antitumor activity [10]. Astragaloside IV, a cycloartane triterpene glycoside extracted from Radix Astragali, has a broad range of pharmacological properties, including antiapoptotic [11], anti-inflammatory and antihypertensive [12] effects.

As part of our efforts to isolate the chemical constituents of Astragali Radix to evaluate *A. membranaceus* qualitatively, we report herein on the isolation of a new minor saponin, agroastragaloside V (**1**), obtained from the roots of *A. membranaceus* cultivated in Korea, together with four known compounds **2**–**5**, and the structural determination of these substances using extensive spectroscopic methods. Several previous studies have provided immune stimulant effects of several cycloartane-type triterpene glycosides and the extracts on macrophage activation and expression of inflammatory cytokines were investigated from *Astragalus* species [9,12]. Therefore, isolated compounds **1**–**5** were evaluated for anti-inflammatory activities through the measurement of nitrite, a soluble oxidation product of nitric oxide (NO), in lipopolysaccharide (LPS)-induced RAW 254.7 macrophage cells.

## 2. Results and Discussion

**Figure 1:**
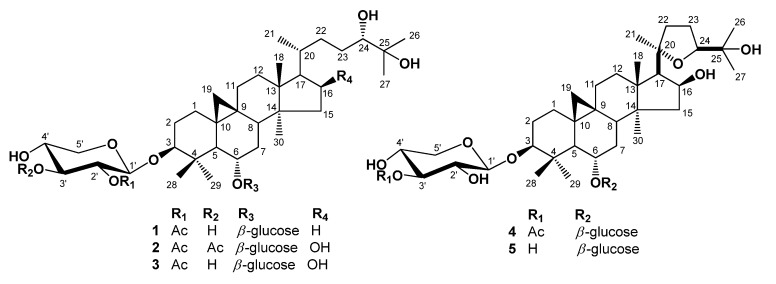
Chemical structures of isolated compounds **1**–**5**.

A 80% methanolic extract of dried roots of *A. membranaceus* was suspended in H_2_O and extracted with EtOAc, and then *n*-BuOH. The EtOAc soluble fraction was concentrated under reduced pressure to produce a residue which was then subjected to multiple chromatographic steps using silica gel, reversed-phase C18, and Sephadex LH-20, yielding the compounds **1**–**5** (Figure 1).

**Figure 2:**
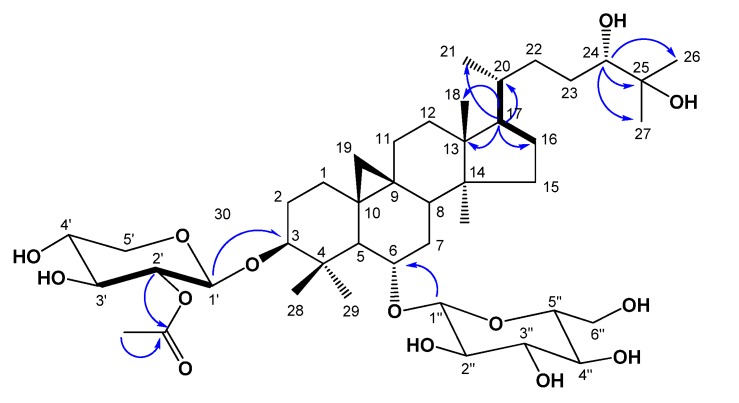
Key ^1^H-^1^H COSY (bold dash) and HMBC (blue arrow) correlations of compound **1**.

Compound **1**, was obtained as an amorphous white powder from MeOH. High-resolution (HR) FAB-MS exhibited an ion peak for [M−H]^−^ at *m*/*z* 811.4777, which is compatible with the molecular formula C_4__3_H_7__2_O_1__4_. The IR spectrum of **1** showed the presence of a hydroxyl group (3433 cm^−1^) and an ester carbonyl group (1,724 cm^−1^). The ^1^H-NMR spectrum of **1** (Table 1) revealed the presence of a cyclopropane methylene group with signals at δ_H_ 0.17 (1H, d, *J* = 4.0 Hz) and 0.53 (1H, d, *J* = 4.0 Hz) and also contained signals for six tertiary methyl groups at δ_H_ 0.95, 1.28, 1.38, 1.41, 1.43, and 1.78, and for one acetyl methyl group (δ_H_ 2.03) which were correlated in HSQC with carbon signals at δ_C_ 19.9, 16.6, 18.6, 25.7, 26.5, 28.3 and acetyl (δ_C_ 21.2), respectively. A secondary methyl group at δ_H_ 1.07 (3H, d, *J* = 6.4 Hz) and at δ_C_ 18.3, and four oxygen bearing methine proton signals at δ_H_ 4.48 (ddd, *J* = 8.0, 8.0, 5.2 Hz), 3.53 (ddd, *J* = 9.5, 9.5, 4.5 Hz), 3.41 (dd, *J* = 10.5, 2.2 Hz) and 3.23 (dd, *J* = 11.3, 4.0 Hz), which were indicative of secondary alcoholic functions (Table 1), were readily noticed in the ^1^H-NMR spectrum. Furthermore, the ^1^H-NMR spectrum of **1** clearly showed two anomeric doublets at δ_H_ 4.77 (*J* = 7.6 Hz), and 4.96 (*J* = 7.2 Hz) in the downfield region, indicating of the presence of two *β*-linked sugars. This was supported by the ^13^C-NMR spectrum, which showed two anomeric carbon signals at δ_C_ 104.7 and 105.2. The chemical shifts of the individual protons of the two sugar units were revealed from a combination of 2D-COSY spectral analyses, and ^13^C chemical shifts of their relative attached carbons were assigned unambiguously from the HSQC and HMBC experiments which led to the identification of a *β*-xylopyranosyl unit and a *β*-glucopyranosyl unit. The sites of attachment of the xylose and glucose moieties of **1** were determined by HMBC experiment to be at C-3 and C-6, respectively. In the HMBC spectrum, the first anomeric proton signal at δ_H_ 4.77 (H-1') showed long range correlation with the carbon at δ_C_ 89.0 (C-3). Also the second anomeric proton signals at δ_H_ 4.96 (H-1") showed long-range correlation with the carbons at δ_C_ 79.1 (C-6), respectively. Consequently, xylose and glucose should be attached to the hydroxyl groups at C-3 and C-6. The relative configuration of OH-6 was confirmed by a NOESY experiment, which showed a correlation between H-6 and H-28, H-19a, as well as H-8. These NOE correlations indicated that the oxygen at C-6 is *α*-oriented. In the ^1^H-NMR spectrum, signals at 2.03 (3H, s), and ^13^C-NMR signals at 170.0, showed the presence of an acetoxyl group in **1**. The NMR data of the acetoxyl moiety of **1** were in good agreement with those reported for agroastagaloside II (**3**) [13,14]. Also, the cross peak between the oxygenated methine proton signal of xylose (H-2') and the ester carbon signal (δ_C_ 170.0) suggested that the acetyl group was linked to the hydroxyl of C-2' of xylose (Figure 2) in the HMBC spectrum. This was confirmed by the down-field shifts of the carbon (δ_C_ 75.7) and proton signal (δ_H_ 5.52, H-2') owing to the esterification effect. The ^13^C-NMR spectrum of **l** displayed a total of 43 carbon signals. Based on a DEPT experiment, the HSQC spectrum, and a comparison with the ^13^C-NMR data of the related agroastagaloside II (**3**), all the signals could be assigned (Table 2). These data were similar to agroastagaloside II (**3**) with the exception of the proton and carbon resonances for the lack of an oxygenated methine moiety at the C-16 position, respectively. The methylene signals due to C-16 in **1** were replaced by those of an oxygenated methine (δ_H_ 4.72 and δ_C_ 72.1) in agroastagaloside II (**3**) [14]. The molecular weight of **1** was 16 Da less than that of agroastagaloside II (**3**), indicating the presence of one less hydroxyl group. This conclusion was also supported by the HMBC spectrum, which showed ^2^*J*, ^3^*J*, and long range correlation between the proton signal of H-17 (δ_H_ 1.51) and the carbon signals of C-13 (δ_C_ 45.8), C-16 (δ_C_ 28.7), C-20 (δ_C_ 28.6), C-18 (δ_C_ 18.6) and C-21 (δ_C_ 18.3) (Figure 2). The d-configurations of xylose and glucose units were established after hydrolysis of **1** followed by GC analysis [15]. Finally, the structure of **1** was determined to be 3-*O*-*β*-(2'-*O*-acetyl)-d-xylopyranosyl-6-*O*-*β*-d-glucopyranosyl-(24*S*)-3*β*,6*α*,24*α*,25-tetrahydroxy-9,19-cyclolanostane, and named agroastragaloside V. Comparisons of NMR and MS data for the known compounds **2**–**5** with reported values led to their identification as agroastragaloside I (**2**) [16], agroastragaloside II (**3**) [14], isoastragaloside II (**4**) [13] and astragaloside IV (**5**) [16], respectively (Figure 1).

**Table 1:** ^1^H- (400 MHz) and ^13^C-NMR (100 MHz) data of compound **1** (in pyridine-*d_5_*, *δ* in ppm, *J* in Hz) ^a^.

No.	δ_H_	δ_C_ (DEPT)	No.	δ_H_	δ_C_ (DEPT)
1	1.27 ^b^, 1.55 ^b^, m	32.1 (CH_2_)	23	1.68 ^b^, 1.96 ^b^, m	27.9 (CH_2_)
2	1.68 ^b^, 1.94 ^b^, m	30.0 (CH_2_)	24	3.91, brd, *J* = 10.8	77.1 (CH)
3	3.39, dd, *J* = 4.4, 11.6	89.0 (CH)	25	-	72.5
4	-	42.3	26	1.43, s	25.7 (CH_3_)
5	1.93, d, *J* = 8.8	52.5 (CH)	27	1.41, s	26.5 (CH_3_)
6	3.78, ddd, *J* = 4.4, 9.6, 9.6	79.1 (CH)	28	1.78, s	28.3 (CH_3_)
7	1.82, 2.25, m	34.5 (CH_2_)	29	1.28, s	16.6 (CH_3_)
8	1.90, m	45.8 (CH)	30	0.95, s	19.9 (CH_3_)
9	-	21.5	1'	4.77, d, *J* = 7.6	104.7 (CH)
10	-	28.7	2'	5.52, dd, *J* = 8.0, 8.0	75.7 (CH)
11	1.15, 1,89 ^b^, m	26.3 (CH_2_)	3'	4.15 ^b^, m	76.3 (CH)
12	1.64 ^b^, 2.35 ^b^, m	33.2 (CH_2_)	4'	4.14 ^b^, m	71.4 (CH)
13	-	45.8	5'	4.27 ^b^, m, H-5'a 3.62, dd, *J* = 9.6, 11.6, H-5'b	67.1 (CH_2_)
14	-	46.9	1''	4.96, d, *J* = 7.2	105.2 (CH)
15	1.45 ^b^, 1.66 ^b^, m	30.0 (CH_2_)	2''	4.00, dd, *J* = 8.0, 8.0	75.6 (CH)
16	1.33 ^b^, 1.54 ^b^, m	28.7 (CH_2_)	3''	4.29, m	79.1 (CH)
17	1.51 ^b^, m	49.7 (CH)	4''	4.10, dd, *J* = 8.8, 8.8	72.0 (CH)
18	1.38, s	18.6 (CH_3_)	5''	3.88, m	78.1 (CH)
19	0.17, d, *J* = 4.0, H-19a 0.53, d, *J* = 4.0, H-19b	28.4 (CH_2_)	6''	4.42, dd, *J* = 2.4, 11.2, H-6''a 4.29, dd, *J* = 3.6, 11.2, H-6''b	63.2 (CH_2_)
20	2.39 ^b^, m	28.6 (CH)	COCH_3_	-	170.0
21	1.07, d, *J* = 6.4	18.3 (CH_3_)	COCH_3_	2.03, s	21.2 (CH_3_)
22	1.40, 1.99 ^b^, m	33.0 (CH_2_)			

^a^ Assignments were confirmed by ^1^H-^1^H COSY, HSQC, and HMBC. ^b^ Signals are unclear due to overlapping.

Previous studies have already reported on the anti-inflammatory effects of components obtained from *A. membranaceus* [9,12]. Thus, we also investigated the inhibitory effects of compounds **1**–**5** on NO production by using the Griess reaction to measure nitrite, a soluble oxidation product of NO, in the culture medium of LPS-induced RAW 264.7 macrophages. As shown in Table 2, compounds **1**–**5** inhibited NO production with IC_50_ values in the range of 1.38 to 4.70 μM, respectively. Some cell toxicity was observed in cells treated with compounds **2**, **3** and **4**, whereas other compounds had no influence on cell viability.

**Table 2:** Inhibitory effects of compounds **1**–**5** against LPS-Induced NO production in RAW 264.7 macrophage cells.

Compound	IC_50_ (μM) ^a^	cell viability (%) ^b^
1	1.85 ± 0.24	93.15 ± 6.96
2	1.38 ± 0.15	54.54 ± 1.21
3	2.31 ± 0.47	47.56 ± 3.40
4	4.70 ± 1.77	68.98 ± 1.82
5	2.09 ± 0.27	94.42 ± 4.33
Caffeic acid ^c^	0.83 ± 1.15	82.20 ± 1.64

^a^ The IC_50_ value of each compound was defined as the concentration (μM) that caused 50% inhibition of NO production in LPS-activated RAW 264.7 macrophage cells. Cells were pretreated for 1 h with compounds before stimulation with LPS (1 μg/mL) for 7 h; ^b^ Cell viability indicates mean maximum inhibitory effect, at a concentration of 100 μM, expressed as a percentage inhibition of nitrite production induced by LPS (1 μg/mL) in the presence of vehicle; ^c^ Positive control. The results are averages of three independent experiments, and the data are expressed as mean ± SD.

## 3. Experimental

### 3.1. General

^1^H-, ^13^C-, and 2D-NMR spectra were recorded on a Varian Unity Inova AS 400 FT-NMR instrument, and the chemical shifts were given in δ (ppm) based on the use of tetramethylsilane (TMS) as an internal standard. Optical rotations were measured on a JASCOP-1010 digital polarimeter. IR spectra were run on a Perkin Elmer Spectrum One FT-IR spectrometer. HR-FABMS spectra were obtained using a JEOL JMS-700 mass spectrometer (Tokyo, Japan). A Shimadzu gas chromatograph (GC-14B) equipped with an on-column injection system and flame ionization detector (FID) was used (Tokyo, Japan). Silica gel 60 (Merck, 230–400 mesh), LiChroprep RP-18 (Merck, 40–63 μm), and Sephadex LH-20 (Amersham Pharmacia Biotech., Uppsala, Sweden) were used for column chromatography (CC). Pre-coated silica gel plates (Merck, Kieselgel 60 F_254_, 0.25 mm) and pre-coated RP-18 F_254s_ plates (Merck) were used for analytical thin-layer chromatography analyses. Spots were visualized by spraying with 10% aqueous H_2_SO_4_ solution followed by heating.

### 3.2. Plant Material

The roots of *A. membranaceus* were cultivated in Jecheon, Chungbuk Province, Korea, for one year, harvested in September 2011, and identified by Dr. Jung-Hun Lee, National Institute of Horticultural and Herbal Science (NIHHS), Rural Development Administration (RDA). A voucher specimen (MPS00874) was preserved at the NIHHS, RDA.

### 3.3. Extraction and Isolation

The roots of *A. membranaceus* (10 kg) were powdered and extracted three times with 36 L of aqueous 80% MeOH at room temperature for 24 h. After concentration *in vacuo*, the MeOH extract (1,387 g) was suspended in H_2_O (3 L) and then partitioned with EtOAc (3 L × 3) followed by concentration to give the EtOAc fraction (E, 57 g). Fraction E (ARE, 57 g) was subjected to a silica gel CC (10 × 21 cm) using a gradient of CH_2_Cl_2_–MeOH–H_2_O (15:3:1→12:3:1→9:3:1→7:3:1→MeOH, each 2.5 L to yield 23 fractions (E1 to E23). Fraction E10 [2.51 g, elution volume/total volume (Ve/Vt) 0.45–0.57] was subjected to the RP-18 silica gel CC [4.5 × 12 cm, MeOH–H_2_O (1:1.8, 2.5 L)] to give compound **2** [184 mg, Ve/Vt 0.43–0.65, (RP-18 F_254s_) R_f_ 0.50, MeOH–H_2_O (5:1)]. Subfraction E17 (400 mg, Ve/Vt 0.66–0.70) was separated by CC [RP-18 (4.5 × 8 cm), MeOH–H_2_O (2.5:1, 1.5 L)] to give compound **4** [25 mg, Ve/Vt 0.38–0.45, TLC (RP-18 F_254s_) R_f_ 0.30, MeOH–H_2_O (3:1)]. Fraction E21 [140 mg, Ve/Vt 0.88–0.92] was subjected to the RP-18 CC [3.5 × 7.5 cm, MeOH–H_2_O (3:1) to give five subfractions (E21-1 to E21-5). Subfraction E21-3 (22 mg, Ve/Vt 0.59–0.68) was fractionated using a Sephadex LH 20 CC [2.5 × 50 cm, MeOH–H_2_O (4:1, 800 L)] and yielded compound **1** [12 mg, Ve/Vt 0.66–0.85, TLC (RP-18 F_254s_) Rf 0.40, MeOH–H_2_O (5:1)]. Subfraction E21-6+7 (45 mg, Ve/Vt 0.59–0.68) was subjected to the RP-18 CC [2.5 × 7.5 cm, MeOH–H_2_O (3:1) to give compound **5** [18 mg, Ve/Vt 0.67–0.90, TLC (RP-18 F_254s_) R_f_ 0.35, MeOH–H_2_O (5:1)]. Fraction E22 [140 mg, Ve/Vt 0.93–0.98] was subjected to the RP-18 CC [3.5 × 6 cm, MeOH–H_2_O (3:1) to give five subfractions (E22-1 to E22-6). Subfraction E22-4 (78 mg, Ve/Vt 0.72–0.81) was fractionated using a RP-18 CC [2.5 × 5 cm, MeOH–H_2_O (2.5:1, 800 L)] and yielded compound **3** [33 mg, Ve/Vt 0.65–0.80, TLC (RP-18 F_254s_) Rf 0.50, MeOH–H_2_O (5:1)].

### 3.4. Spectroscopic Data

*Agroastragaloside V* (**1**). Amorphous white powder; 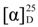 −18.5° (*c* = 0.15, MeOH); IR (CaF_2_ window) cm^−1^: 3433, 1724, 1510, 1245, 1065; HR-FAB/MS *m*/*z* 811.4777 [M−H]^−^ (calcd for C43H71O14, 811.4843); ^1^H- and ^13^C-NMR data, see Table 1.

### 3.5. Acid Hydrolysis and GC Analysis

A solution of compound **1** (3 mg) in 2 N HCl (2 ml) was heated at 80 °C for 6 h. The mixture was cooled at 0 °C and neutralization with 2 N NaOH in H_2_O (2 mL) and then extracted with CHCl_3_. The aqueous layer was concentrated under a vacuum to give a residue of the sugar fraction. The residue was dissolved in pyridine (100 μL), and then 0.1 M L-cysteine methyl ester hydrochloride (150 μL) was added. After reacting at 60 °C for 90 min, the reaction mixture was dried under a vacuum. For derivatization, 100 μL of *N*-methyl-*N*-(trimethylsilyl)trifluoroacetamide (MSTFA) was added and the mixture incubated at 37 °C for 30 min. Then, the mixture was subjected to GC analysis under the following conditions: capillary column, DB-5 (30 m × 0.32 mm × 0.25 μm); detector, FID; detector temperature, 280 °C; injector temperature, 250 °C; carrier, N_2_ gas (20.4 mL/min); oven temperature, 170–250 °C with a rate of 5 °C/min, with one μL of each sample injected directly into the inject port (split-less mode). The peaks from the hydrolyrate of **1** were detected at 9.24 and 10.02 (d-xylose) and 12.66 min d-glucose). The retention times for authentic samples in the same experimental conditions were detected at 9.24 and 10.02 (d-xylose, Sigma), and 12.66 min (d-glucose, Sigma), respectively.

### 3.6. Measurement of NO Production and Cell Viability

Assays for NO production and cell viability were carried out as previously described [17]. Briefly, RAW 264.7 macrophages were harvested and seeded in 24-well plates (3 × 10^5^ cells/well) for the measurement of NO production. The plates were pretreated with various concentrations of samples for 1 h and incubated with LPS (1 μg/mL) for 7 h. The amount of NO was determined by the nitrite concentration in the cultured RAW264.7 macrophage supernatants using the Griess reagent. The cell viability was evaluated by MTT reduction.

## 4. Conclusions

The new compound 3-*O*-*β*-(2'-*O*-acetyl)-d-xylopyranosyl-6-*O*-*β*-d-glucopyranosyl-(24*S*)-3*β*,6*α*, 24*α*,25-tetrahydroxy-9,19-cyclolanostane, named agroastragaloside V (**1**), was isolated from *Astragalus membranaceus*, together with four known cycloartane glycosides. According to previous investigations of the various *Astragalus* species, we have evaluated the inhibitory activities of all compounds against LPS-induced NO production in RAW264.7 macrophages. Agroastragaloside V and astragaloside IV showed significant inhibition of NO production without cytotoxicity. The results provide a potential explanation for the use of this plant as a herbal medicine in the treatment of inflammatory diseases, and they merit consideration as leads for anti-inflammatory agents.

## Supplementary Materials

^1^H-NMR, ^13^C-NMR, and HR-FABMS spectra of **1** are available free of charge via the internet at http://www.mdpi.com/1420-3049/18/4/3725/s1.
