# Supplementary material for: Anti-Inflammatory Cycloartane-Type Saponins of Astragalus membranaceus
Source: Molecules. 2013 Mar 25;18(4):3725–32. doi: 10.3390/molecules18043725 (PMC6269660; doi:10.3390/molecules18043725)

Supplementary Materials

Figure S1. ^{1}H-NMR of compound **1**.

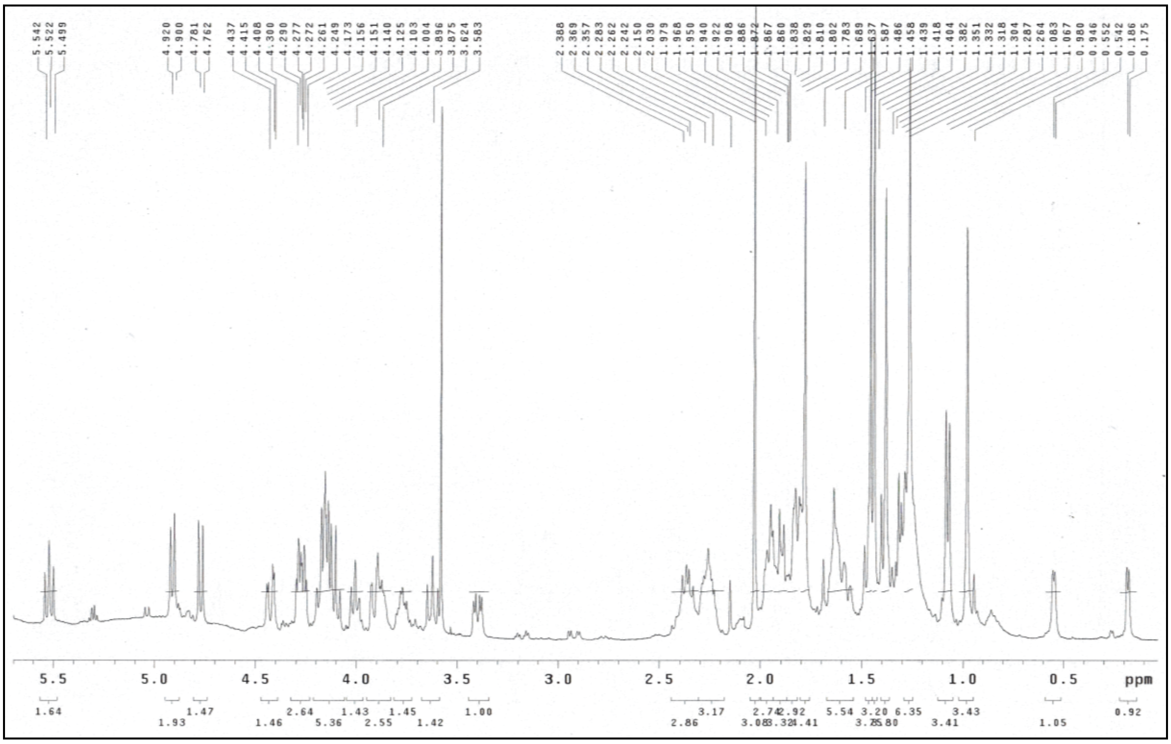

Figure S2. ^{13}C-NMR of compound **1**.

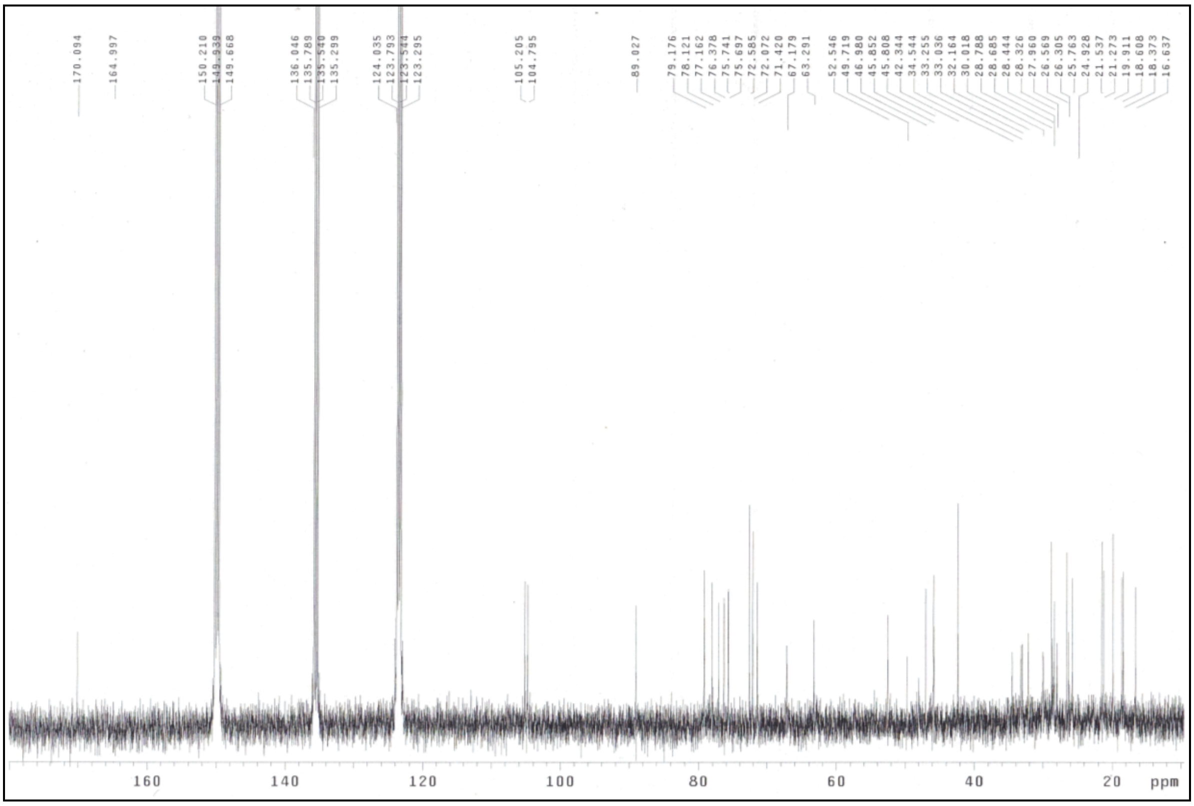

Figure S3. HR-FABMS of compound **1**.

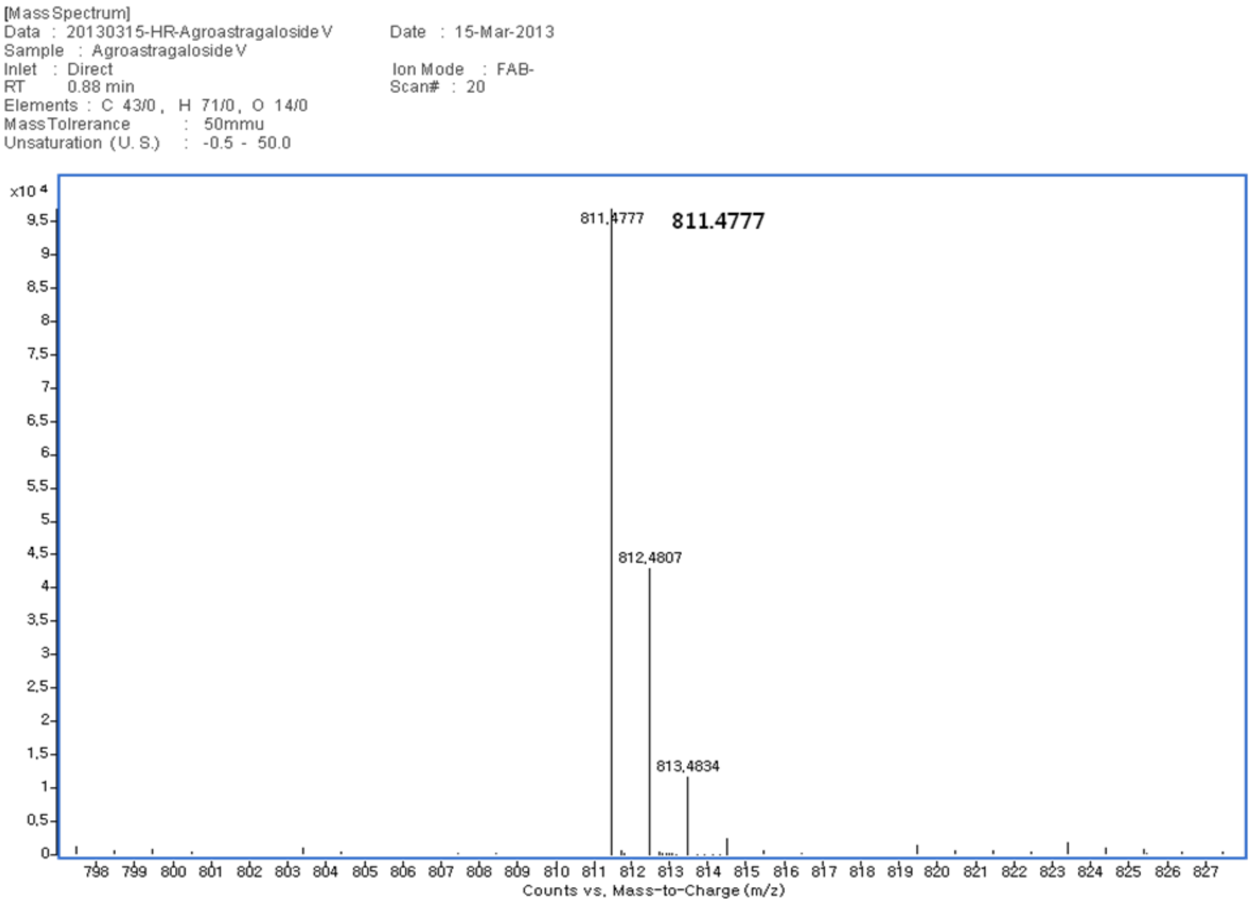

Supplement: Supplementary file 1 [file molecules-18-03725-s001.pdf]
